# *Hermetia illucens* Larvae Meal Enhances Colonic Antimicrobial Peptide Expression by Promoting Histone Acetylation in Weaned Piglets Challenged with ETEC in Pig Housing

**DOI:** 10.3390/ani16010118

**Published:** 2025-12-31

**Authors:** Qingsong Tang, Guixing Wu, Wentuo Xu, Jingxi Liu, Huiliang Liu, Bin Zhong, Qiwen Wu, Xuefeng Yang, Li Wang, Zongyong Jiang, Hongbo Yi

**Affiliations:** State Key Laboratory of Swine and Poultry Breeding Industry, Key Laboratory of Animal Nutrition and Feed Science in South China, Ministry of Agriculture and Rural Affairs, Guangdong Provincial Key Laboratory of Animal Breeding and Nutrition, Institute of Animal Science, Guangdong Academy of Agricultural Sciences, Guangzhou 510642, China; tqs5213@foxmail.com (Q.T.);

**Keywords:** *H. illucens* larval meal, ETEC, immune homeostasis, histone acetylation, weaned piglets

## Abstract

The objective of this study was to investigate the effects of replacing fishmeal with *H. illucens* larval meal on the colonic immune homeostasis in weaned piglets in enterotoxigenic *Escherichia coli* (ETEC)-challenged pig housing. Seventy-two weaned piglets, aged 28 days, were randomly divided into three groups for dietary treatment: the basal diet (negative control, NC), the positive control diet (PC) supplemented with 1445 mg zinc/kg zinc oxide in the basal diet, and the *H. illucens* larval meal complete replacement of fishmeal in the basal diet (HILM), for 28 days in ETEC-challenged pig housing. The results showed that the relative transcript abundances of *ZO-1*, *pBD2*, *PR39*, and *PG1–5* were increased (*p* < 0.05) in pigs fed the HILM diet compared with those fed the NC diet. In addition, the HILM diet reduced (*p* < 0.05) the serum contents of IL-8 and increased (*p* < 0.05) the serum contents of IL-10 and IgG compared with the NC diet. In terms of the molecular mechanisms by which immune homeostasis is improved, the p-NF-κB/ NF-κB ratio and TLR2 protein expression in the colon were decreased (*p* < 0.05) in pigs fed the HILM diet compared with those fed the NC diet. Compared with the NC diet, the HILM diet reduced (*p* < 0.05) the protein expression of HDAC3 and HDAC7 in the colon of pigs. The SIRT1, acH3K9, and pH3S10 protein expressions in the colon were the greatest (*p* < 0.05) in pigs fed the HILM diet compared with the NC diet. HILM diets improved the colonic immune homeostasis in weaned piglets by enhancing the antimicrobial peptide expression, thereby mitigating ETEC challenges in pig housing. Mechanistically, HILM diets promote antimicrobial peptide expression through increased histone acetylation (acH3K9 and pH3S10).

## Simple Summary

Population growth and climate change pose challenges to the sustainability of pig farming. The focus on new sources of protein feedstuffs for piglets is receiving increasing attention. *H. illucens* larvae meal may not only be high in protein but may also play an important role in the resistance to bacterial infections and immunomodulation. In this study, we investigate the effects of *H. illucens* larval meal on the colonic immune homeostasis of weaned piglets and further investigate the molecular mechanism in an ETEC K88-challenged environment. The results of this experiment may provide new insights for *H. illucens* larvae meal as protein feedstuffs and for improving the immune homeostasis of piglets.

## 1. Introduction

The global population is growing, which has led to a dramatic increase in the demand for protein feed resources for pig farming [1]. Fishmeal is an important source of protein in piglets’ feedstuffs. However, the price of fishmeal is rising due to overfishing and climate issues, reducing the sustainable use of fishmeal [2]. China is the largest consumer of fishmeal in the world. The total production of fishmeal is far from meeting the demand of pig farming, with an annual import dependency of about 72% [3]. Therefore, there is an urgent need to find a new source of protein feedstuffs that is safe, effective, and sustainable. *Hermetia illucens* (*H. illucens*) larvae are able to produce high levels of crude protein (35–48%), crude fat (10–39%), and vitamins and minerals by breaking down food processing by-products and kitchen leftovers, thus enabling a “circular bioeconomy” [4]. In addition, the amino acid composition of *H. illucens* larvae meal is similar to fishmeal and has a high amino acid transport capacity in the animal [2,5]. Therefore, *H. illucens* larvae may be a potential source of protein feedstuffs for pig farming.

Research has shown that pigs’ feeding diets containing 2% or 4% *H. illucens* larvae meal reduce the expression of tumor necrosis factor-alpha (TNF-α) and increase the expression of interleukin-10 (IL-10) in their intestinal mucosa [6,7]. In the past few years, studies have shown that *H. illucens* larvae are rich in beneficial biomass such as antimicrobial peptides (AMPs), lauric acid, adipic acid, chitin, and chitosan, all of which play an important role in the resistance to bacterial infections and immunomodulation [8,9]. Enterotoxigenic *Escherichia coli* (*E. coli*) K88 (ETEC K88)-induced diarrhea is accompanied by the increased secretion of intestinal inflammatory cytokines and reduced expression of tight junction proteins, resulting in an immune imbalance in piglets [10]. ETEC K88 bind to cell surface receptors and stimulate intestinal epithelial cells and macrophages to activate nuclear factor-κB (NF-κB) signaling pathways, exacerbating intestinal inflammation [11]. The AMPs and chitosan extracted from *H. illucens* have a strong inhibitory effect on ETEC K88 [12,13]. Therefore, *H. illucens* larvae meal may not only be used as a new protein source for feedstuffs, but may also regulate the intestinal immune homeostasis of weaned piglets. However, in ETEC-challenged pig housing, the effects of replacing fishmeal with *H. illucens* larvae meal on the colonic immune homeostasis of weaned piglets is unclear to date.

Two classical pathways associated with the immune response and that are important to processes such as inflammation regulation and cell survival are NF-κB and mitogen-activated protein kinase (MAPK) signaling pathways [14,15]. Histone acetylation modifications specifically regulate the transcriptional expression of AMPs. Increased expression levels of acetylation of histone 3 lysine 9 (acH3K9), acH3K27, acH3K56, and histone H3 at serine s10 (pH3S10) are typical markers of the transcriptional activation of AMPs [16]. Previous studies have shown that feeding *H. illucens* larvae meal can reduce the NF-κB gene expression in weaned piglets and fattening pigs [6,7]. However, the molecular mechanisms by which feeding *H. illucens* larval meal regulates the NF-κB /MAPK signaling pathway and histone acetylation modifications in response to the immune homeostasis of weaned piglets in ETEC-challenged pig housing have rarely been reported.

Thus, the aim of this study was to investigate the effects of *H. illucens* larval meal replacement fishmeal on the colonic immune homeostasis of weaned piglets, and further investigate the molecular mechanism in ETEC-challenged pig housing.

## 2. Materials and Methods

### 2.1. Animal Treatment

*H. illucens* larvae meal is sourced from Guangzhou AnRuiJie Environmental Protection Technology Co., Ltd. (Guangzhou, China). A total of 72 weaned piglets (Duroc × Landrace × Large White) with initial weight of 8.44 ± 0.04 kg were randomly divided into three treatments: the negative control treatments (NC) were fed a basal diet containing 3% fishmeal; the positive control treatments (PC) were supplemented with 1445 mg zinc/kg zinc oxide in the basal diet; and the *H. illucens* larvae meal treatments diet (HILM) used *H. illucens* larvae meal as a complete replacement for fishmeal in the basal diet. Each treatment consisted of six replicates, with four pigs per replicate. The experimental diets were formulated according to National Research Council (NRC2012) nutrient recommendation (Table S1). All pigs were provided the indicated experimental diet and water ad libitum during the 28-day experiment.

### 2.2. Simulation of K88 Challenged Environment

The frozen ETEC K88 strain was thawed and added to 10 mL LB broth and incubated for 12 h at 37 °C on a shaker. The working bacterial solution (6 × 10^8^ CFU/mL) was sprayed on days 1, 5, 9, 13, 17, 21, and 25 of the experiment. An ultra-low-volume sprayer (Yitaizheng 211) was used to apply a bacterial solution containing 6 × 10^8^ CFU/mL of ETEC K88 in the pig housing. A total of 2 L bacterial solution was sprayed throughout the pig housing, with the equipment set to a constant flow rate to ensure uniform distribution. On day 10 of the experiment, three 5 × 5 cm areas in the leaking fecal platforms and aisles, as well as three drinking fountains, were selected for environmental sample collection. The environmental samples were quickly placed in ice boxes and brought back to the laboratory for incubation and detection.

### 2.3. Sample Collection

At the end of the experiment, one pig was randomly selected from each replicate for blood collection from the anterior vena cava. Then, one pig was randomly selected from each replicate and euthanized after intravenous administration of sodium pentobarbital solution (40 mg/kg body weight). The heart, liver, spleen, lungs, and kidneys were weighed for the relative weight (% of body weight) of organs (relative weight of organs [%] = organ weight/body weight × 100). The contents of colon were collected carefully. The segments of colon were meticulously opened and thoroughly rinsed with sterile normal saline and then fixed in 4% paraformaldehyde for intestinal morphology analysis. The colonic tissue was collected and stored at −80 °C for further analysis.

### 2.4. DNA Extraction and Measurement of Intestinal Bacterial Populations by Quantitative Real-Time PCR (qRT-PCR)

The extraction of bacterial DNA from the colonic contents was conducted in accordance with the manufacturers’ instructions of the HiPure Stool DNA Kit B (Magen, Guangzhou, China). The specific primers of total bacteria (forward primer: CGGTGAATACGTTCYCGG; reverse primer: GGWTACCTTGTTACGACTT) [17] and *E. coli* (forward primer: CATGCCGCGTGTATGAAGAA; reverse primer: CGGGTAACGTCAATGAGCAAA) [18] were commercially synthesized from Sangon Biotechnology Co., Ltd. (Shanghai, China). The number of total bacteria and *E. coli* were analyzed by qRT-PCR using SYBR Green method and the CFX96 Real-Time PCR Detection System (Bio-Rad, Hercules, CA, USA) as previously described [6]. Each 20 μL reaction included 10 μL iTaq Universal SYBR Green Supermix (Bio-Rad, Hercules, CA, USA), 2 μL DNA, 0.4 μL forward primer (10 μM), 0.4 μL reverse primer (10 μM), and 7.2 μL ddH_2_O. Additionally, bacterial copies were transformed (log10) before statistical analysis.

### 2.5. Serum Cytokines and Immunoglobulin Determined by ELISA

Next, 100 mg of tissue sample was added to 900 μL of 0.86% saline and homogenized, and then spun at 3500× *g* at 4 °C for 10 min. The supernatant was then determined. The concentrations of IL-1β, IL-6, IL-8, IL-10, TNF-α, transforming growth factor-β (TGF-β), IgA, IgG, IgM, diamine oxidase (DAO), and D-Lactic acid (D-LA) were determined in duplicate using porcine ELISA kit (Mlbio, Shanghai, China). All procedures were performed according to the manufacturers’ instructions.

### 2.6. Quantitative Real-Time PCR

The total RNA was isolated from the colonic tissue with TRIzol reagent (Thermo Fisher Scientific, Waltham, MA, USA), and RNA quality was measured using Nanodrop 1000 spectrophotometer (Thermo Fisher Scientific, Waltham, MA, USA). Subsequently, 1000 ng RNA was reverse-transcribed to cDNA using a Synthesis Kit (Takara, Dalian, China). Real-time PCR of target genes was performed using the SYBR Green method. The reaction mixtures (10 μL) were set according to a previous study [3]. All primers were commercially synthesized from Sangon Biotechnology Co., Ltd. (Shanghai, China) and listed in Table S2. The 2^−ΔΔCt^ method was used to analyze the relative mRNA expression of the target genes, and the data for each target gene were normalized to the NC pigs.

### 2.7. Western Blot

Total protein was extracted from colonic tissue using RIPA buffer (Biosharp, Hefei, China) containing 1% protease inhibitor cocktail and phosphatase inhibitor (Apexbio, Houston, TX, USA). Approximately 30 μg denatured proteins were separated using 10% SDS-PAGE (Bio-Rad, Hercules, CA, USA) and transferred onto PVDF membranes. The membranes were incubated with appropriate primary antibodies overnight at 4 °C after blocked with 5% bovine serum albumin for 1 h. The antibody information is shown in Table S3. The protein bands were visualized using a Clarity Western ECL Substrate (Bio-Rad, Hercules, CA, USA).

### 2.8. Immunofluorescence

Immunofluorescence was performed using paraformaldehyde-fixed and paraffin-embedded colonic sections, as described [19]. In brief, sections of 5 μm thickness were deparaffinized and rehydrated, then subjected to antigen retrieval. Sections were closed in bovine serum protein at 3% for 30 min, and the blocking solution was discarded and diluted primary antibody (SIRT1, Abcam, UK) was added overnight at 4 °C; then, secondary antibody (goat antirabbit IgG, Abcam, Cambridge, UK) was incubated for 50 min. Nuclei were stained with DAPI. Image acquisition using a laser confocal scanning microscope (Zeiss, Oberkochen, Germany).

### 2.9. Statistical Analyses

Data were checked for normality using Shapiro–Wilk test with SPSS 26 software (SPSS, Chicago, IL, USA). The statistical analyses using one-way ANOVA and Duncan’s multiple-range post hoc test. All quantifications are presented as means and SEM (standard error of mean), with a statistically significant difference identified as a value of *p* < 0.05.

## 3. Results

### 3.1. The Number of E. coli in Environment and Colonic Digesta of Weaned Piglets in ETEC-Challenged Pig Housing

**Figure 1:**
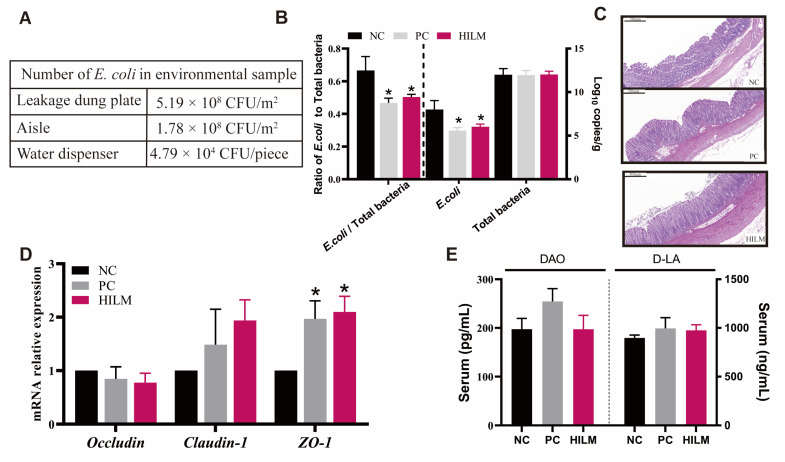
Effect of dietary HILM on the *E. coli* population in pig housing and colonic digesta and colonic barrier function of weaned piglets in ETEC-challenged pig housing. (**A**) The number of *E. coli* in leakage dung plate, aisle, and water dispenser. (**B**) The total bacteria and *E. coli* population (log10 gene copies/g digesta sample) were detected using qRT-PCR, and the ratio of *E. coli* to total bacteria was calculated. (**C**) Representative images of colonic HE stains. (**D**) Relative mRNA expression levels of *occludin*, *claudin-1*, and *ZO-1* in the colon of weaned piglets were detected using qRT-PCR. (**E**) The contents of DAO and D-LA in the serum were detected using ELISA. Data were presented as mean and SEM (*n* = 6). * *p* < 0.05 compared with NC.

The number of *E. coli* were detected at 5.19 × 10^8^ CFU/m^2^, 1.78 × 10^8^ CFU/m^2^ and 4.79 × 10^4^ CFU/piece in the leakage dung plate, aisle, and water dispenser, respectively (Figure 1A). The effects of HILM diet on the *E. coli* population in the colonic digesta of weaned piglets are shown in Figure 1B. The copies of *E. coli* and the ratio of *E. coli* to total bacteria in colonic digesta were reduced (*p* < 0.05) in pigs fed both the HILM and PC diets compared with the NC diet.

### 3.2. Effect of Dietary HILM on Relative Weight of Organ and Colonic Barrier Function of Weaned Piglets in ETEC-Challenged Pig Housing

**Figure 2:**
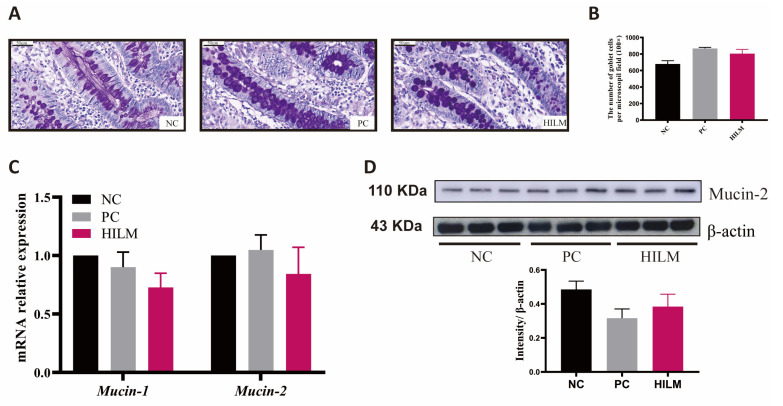
Effect of dietary HILM on mucin secretion in the colon of weaned piglets in ETEC-challenged pig housing. (**A**) Representative images of colonic PAS stains (×500) and (**B**) the number of goblet cells per microscopic field (original magnification ×100). (**C**) Relative mRNA expression levels of *mucin-1*, and *mucin-2* in the colon of weaned piglets were detected by qRT-PCR. (**D**) Protein expression of mucin-2 in the colon of weaned piglets was detected by Western blot and the bar graphs showed the protein band intensity. Data were presented as mean and SEM (*n* = 3).

**Figure 3:**
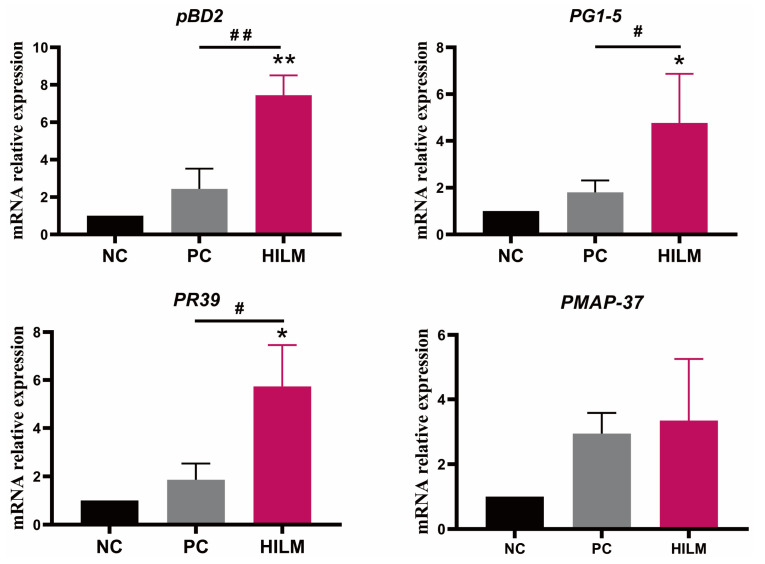
Effect of dietary HILM on AMP expression in the colon of weaned piglets in ETEC-challenged pig housing. Data were presented as mean and SEM (*n* = 6). * *p* < 0.05, ** *p* < 0.01 compared with NC, ^#^ *p* < 0.05, ^##^ *p* < 0.01 HILM compared with PC.

As shown in Table 1, both HILM and PC diets reduced (*p* < 0.05) the relative weight of liver compared with the NC diet. Both the HILM and PC diets improved the colonic morphology (Figure 1C), but did not affect (*p* < 0.05) the number of goblet cells in the colon (Figure 2A,B). Additionally, both the HILM and PC diets increased (*p* < 0.05) the expression of *ZO-1* in the colon compared with the NC diet (Figure 1D). There was no significant difference in the DAO and D-LA in the serum of weaned piglets among the three groups (*p* < 0.05) (Figure 1E). The relative transcript abundance of *mucin-1* and relative transcript abundance and protein expression of mucin-2 was not affected (*p* < 0.05) by dietary treatment (Figure 2C,D). In terms of the AMP expression, the relative transcript abundances of porcine β defensin 2 (*pBD2*), proline-arginine rich 39-amino acid peptide (*PR39*), and protegrin 1–5 (*PG1–5*) were increased (*p* < 0.05) in pigs fed the HILM diet compared with both the NC and PC diets (Figure 3). 

### 3.3. Effects of Dietary HILM on Cytokine Production and Immunoglobulin of Weaned Piglets in ETEC-Challenged Pig Housing

**Figure 4:**
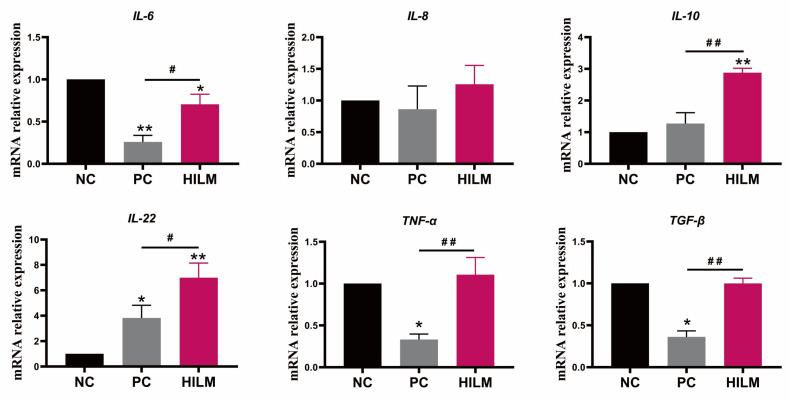
Effect of dietary HILM on cytokine expression in the colon of weaned piglets ETEC-challenged pig housing. Data were presented as mean and SEM (*n* = 6). * *p* < 0.05, ** *p* < 0.01 compared with NC, ^#^ *p* < 0.05, ^##^ *p* < 0.01 HILM compared with PC.

The HILM diet reduced (*p* < 0.05) the contents of IL-8 in the serum and the PC diet reduced (*p* < 0.05) content of IL-1β in the serum compared with the NC diet (Table 2). Both the HILM and PC diets increased (*p* < 0.05) the content of IL-10 in the serum compared with the NC diet. In addition, the concentration of IgG in the serum were increased (*p* < 0.05) in pigs fed the HILM diet and the concentration of IgM in the serum were increased (*p* < 0.05) in pigs fed the PC diet compared with the NC diet. With regard to colonic cytokines, the transcript of *IL-6* in the colon was decreased (*p* < 0.05) and transcripts of *IL-10* and *IL-22* in the colon were increased (*p* < 0.05) in pigs fed the HILM diet compared with the NC diet (Figure 4). Additionally, the PC diet decreased (*p* < 0.05) the transcripts of *IL-6*, *TNF-α*, and *TGF-β* in the colon compared with the NC diet. Meanwhile, the transcripts of *TGF-β*, *IL-10*, *TNF-α*, and *IL-6* in the colon in pigs fed the PC diet were lower (*p* < 0.05) than the HILM diet.

### 3.4. Effects of Dietary HILM on Immune Response and Histone Acetylation Modifications of Weaned Piglets in ETEC-Challenged Pig Housing

**Figure 5:**
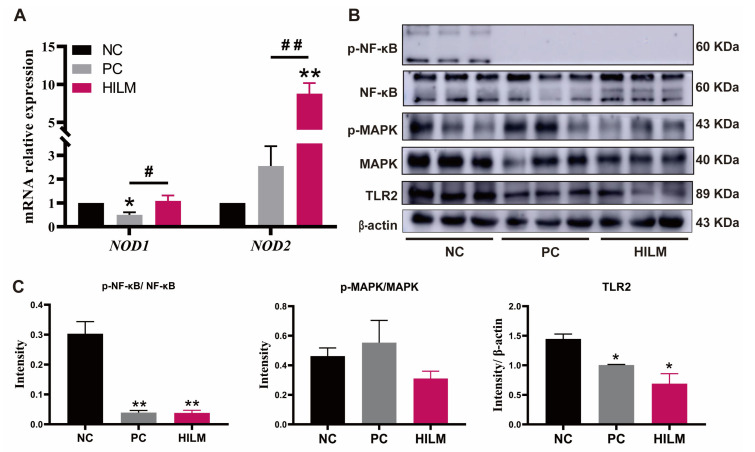
Effect of dietary HILM on NF-κB/MAPK signaling pathway of weaned piglets in ETEC-challenged pig housing. (**A**) Relative mRNA expressions levels of *NOD1* and *NOD2* in the colon of weaned piglets were detected by Real-Time PCR. Protein expressions of p-NF-κB, p-MAPK, and TLR2 in the colon of weaned piglets were detected by Western blot (**B**) and the bar graphs showed the protein band intensity (**C**). Data were presented as mean and SEM (protein expression *n* = 3; others *n* = 6). * *p* < 0.05, ** *p* < 0.01 compared with NC, ^#^ *p* < 0.05, ^##^ *p* < 0.01 HILM compared with PC.

To explore the possible mechanisms by which dietary HILM influences the intestinal immune homeostasis, the relative transcript abundances of nucleotide-binding oligomerization domain 1 (*NOD1*) and nucleotide-binding oligomerization domain 1 (*NOD2*), and the key protein abundance of the NF-κB/MAPK pathway was examined in the colon of weaned piglets. Compared with both the NC and PC diets, the HILM diet increased (*p* < 0.05) the relative transcript abundance of *NOD2* in the colon of weaned piglets (Figure 5A). However, the relative transcript abundance of *NOD1* in the colon was decreased (*p* < 0.05) in pigs fed the PC diet compared with both the NC and HILM diets. Compared with the NC diet, the HILM diet decreased the TLR2 protein expression and p-NF-κB/NF-κB ratio in the colon of pigs (Figure 5B,C).

**Figure 6:**
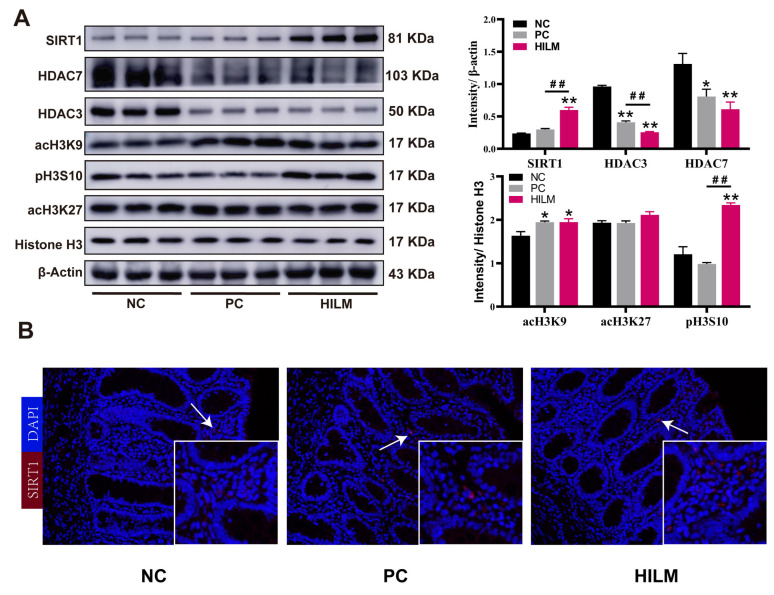
Effect of dietary HILM on histone acetylation modifications of weaned piglets in ETEC-challenged pig housing. (**A**) Protein expressions of SIRT1, HDAC7, HDAC3, acH3K9, acH3K27, and pH3S10 in the colon of weaned piglets was detected by Western blot and the bar graphs showed the protein band intensity. (**B**) The SIRT1 expression in the colon confirmed using immunofluorescence. Red, SIRT1; blue, DAPI; 200×. Data were presented as mean and SEM (protein expression *n* = 3, others *n* = 6). * *p* < 0.05, ** *p* < 0.01 compared with NC, ^##^ *p* < 0.01 HILM compared with PC.

Histone acetylation modification is one of the important molecular mechanisms regulating the transcriptional expression of AMPs. Given the roles of AMPs in intestinal immunity and barrier function, the effects of the dietary HILM diet on histone acetylation modification was examined. The protein expression of SIRT1 in the colon was increased (*p* < 0.05) in pigs fed the HILM diet compared with the NC and PC diets (Figure 6A). These results on the colonic SIRT1 expression with HILM diets were confirmed at the protein level by immunofluorescence (Figure 6B). Compared with the NC diet, both the HILM and PC diets reduced (*p* < 0.05) the protein expressions of HDAC3 and HDAC3 in the colon of pigs. In addition, the protein expression acH3K9 in the colon was the greatest (*p* < 0.05) in pigs fed the HILM and PC diets compared with the NC diet, and the protein expression of pH3S10 in the colon of pigs fed the HILM diet was higher (*p* < 0.05) than that of pigs fed either the NC or PC diet.

## 4. Discussion

**Figure 7:**
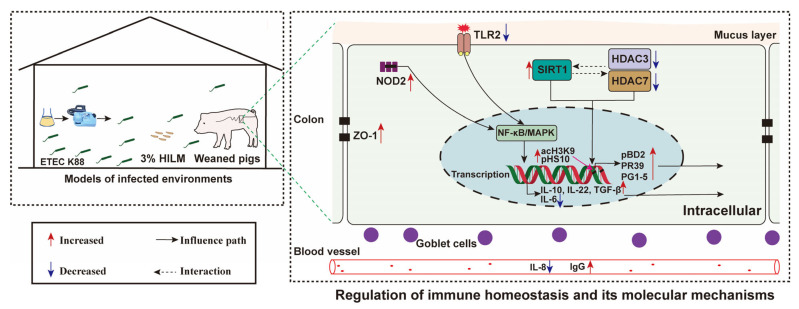
Mechanisms of HILM improving immune homeostasis in weaned piglets. Red arrows indicate an increase in the value of the parameter detected compared to NC; blue indicates a decrease.

*H. illucens* larvae contains high levels of crude protein, and are rich in beneficial biomass such as AMPs, adipic acid, lauric acid, and chitin, as a potential source of protein feedstuffs for pig farming [20]. In recent years, *H. illucens* larvae meal has emerged as a promising alternative to fishmeal for various applications in animal nutrition. Its use as a feedstuff for piglets, broiler chickens, and aquatic animals has shown remarkable potential in enhancing intestinal health [7,21,22]. In the present study, we found that the replacement of fishmeal with *H. illucens* larval meal improved immune homeostasis by regulating the expression of cytokines, tight junction proteins, and AMPs of weaned piglets in ETEC-challenged pig housing (Figure 7). These positive results suggest that *H. illucens* larvae may be a novel protein feedstuffs source and act as a potential beneficial biomass that improves the intestinal immune homeostasis of weaned piglets. 

In the present study, the experiment sprayed 6 × 10^8^ CFU/mL of ETEC K88 to simulate an infected environment and the number of *E. coli* were detected at 5.19 × 10^8^ CFU/m^2^, 1.78 × 10^8^ CFU/m^2^, and 4.79 × 10^4^ CFU/piece in the leakage dung plate, aisle, and water dispenser, respectively. We also found that the *H. illucens* larvae meal diet similar to the zinc-oxide-supplemented diet reduced the copy numbers (log10 gene copies/g digesta sample) of *E. coli* in the colonic digesta. In the past few years, many studies have shown that AMPs and chitosan extracted from *H. illucens* have a strong inhibitory effect on *E. coli* [12,13,23]. And the AMPs of *H. illucens* larvae had a significant inhibitory effect on pathogenic bacteria such as *E. coli* [24]. This may be one of the reasons why feeding *H. illucens* larvae meal reduces the *E. coli* population in the colonic digesta of weaned piglets. Likewise, a study showed that feeding 2% *H. illucens* larvae meal reduced the copy numbers of *E. coli* in the gut of weaned piglets [7]. *H. illucens* larvae have an important defense mechanism with the help of cysteine protease during an *E. coli* challenge [25]. This suggests that dietary *H. illucens* larvae meal reduced the *E. coli* population in the colonic digesta of weaned piglets in ETEC-challenged pig housing.

The intestinal epithelial barrier serves as the first line of defense against toxins and pathogenic microorganisms in the intestinal lumen and antigenic substances in feed. Tight junction proteins play a critical role in maintaining the structural integrity of the intestinal epithelium. These proteins bind to the cytoskeleton, a structural component of cells, and are considered important regulators of paracellular permeability, the movement of molecules across a cell membrane [26]. In the present study, dietary *H. illucens* larvae meal improved the colonic morphology, and increased the expression of *ZO-1* in the colon of weaned piglets, similar to those fed zinc-oxide-supplemented diets. A previous report showed that feeding 1%, 2%, and 4% *H. illucens* larvae meal to replace fishmeal increased the mRNA expression of *ZO-1*, *occludin,* and *claudin-2* in the intestine of weaned piglets in a quadratic and linear manner [7]. Our previous report showed that dietary *H. illucens* larvae meal also increased the claudin-1 and occludin expression in small intestinal [3]. However, in this study, feeding *H. illucens* larvae meal did not alter the number of goblet cells and mucin expression in the colon of weaned piglets. Previous studies have shown that feeding *H. illucens* larvae meal increases the expression of *mucin-1* and *mucin-2* in the jejunum and ileum of weaned piglets, but does not alter the expression of *mucin-2* in the colon of fattening pigs [3,6,7]. This suggests that *H. illucens* larvae meal may not affect the mucin secretion in the colon of pigs. Overall, dietary *H. illucens* larvae meal increased the tight junction protein expression, which enhanced the colonic barrier function of pigs.

AMPs, a critical component of the specific immune function, as another line of defense against intestinal toxins and pathogenic microorganisms [27]. β-defensins inhibit the ETEC-induced activation of the TLR4-NF-κB signaling pathway and reduce the expression of intestinal mucosal inflammatory cytokines IL-1β and TNF-α [28]. PR39 was originally identified from the intestine of pigs and is secreted mainly by neutrophils and macrophages and has specific antibacterial activity against a wide range of gram-negative bacteria [29]. AMPs such as porcine myeloid antimicrobial peptides-37 (PMAP-37) and PG1–5 have been shown to have antibacterial activity and immunomodulatory effects on a variety of pathogenic bacteria [30]. Research has previously indicated that AMPs can reduce THE intestinal epithelial cell damage in weaned and infected pigs with diarrhea [19]. However, studies on the effect of dietary *H. illucens* larvae meal on the expression of colonic AMPs of weaned piglets have rarely been reported. In the present study, we found that dietary *H. illucens* larvae meal increased the pBD2, PR39, and PG1–5 expression in the colon of weaned piglets. A recent study evaluated the antimicrobial activity and immunomodulatory effects of 36 in vitro synthesized AMPs from *H. illucens* larvae, of which Hill-Cec1 and Hill-Cec10 significantly inhibited ETEC [31]. Grain-based diets increased the expression of endogenous AMPs in *H. illucens* larvae [24]. According to our study, piglets fed with *H. illucens* larvae meal demonstrate an increased expression of colonic AMPs and improved immune homeostasis, which can be attributed to the richness of AMPs found in *H. illucens*.

Weaned piglets are highly susceptible to infection by pathogenic bacteria, resulting in diarrhea and death, as their immune system, intestinal development, and their barrier function are still incomplete. ETEC K88 is the most common pathogen-causing immune disorders in piglets [32]. Shiga toxin and lipopolysaccharide produced by ETEC bind to cell surface receptors and stimulate intestinal epithelial cells and macrophages to activate multiple immune-signaling pathways, releasing large amounts of inflammatory cytokines [11,33]. In the present study, dietary *H. illucens* larvae meal decreased the IL-8 and IgG contents, and increased the IL-10 contents in the serum. Meanwhile, the *H. illucens* larvae meal decreased the IL-6 expression and increased the IL-10 and IL-22 expression in the colon of weaned piglets. IL-10 and IL-22 are produced by the activated natural killer cell and T lymphocyte cell and initiate an innate immune response against the pathogen invasion, particularly in intestinal epithelial cells [34]. Research has indicated that feeding *H. illucens* larvae meal diets can reduce the TNF-α expression and increase the IL-10 expression in the intestine of pigs [6,7]. In the past few years, many studies have shown that *H. illucens* larvae meal is rich in beneficial biomass such as AMPs, lauric acid, adipic acid, chitin, and chitosan, which play an important role in fighting bacterial infections and immunomodulation [8,9]. The improvement in immune function in piglets may be related to these probiotic substances. Of note, the addition of zinc oxide to the basal diet in this experiment reduced the serum IL-1β and increased the IL-10 and IgM levels of weaned piglets, and the expression of colonic IL-6, TNF-α, and TGF-β was lower than in pigs fed the basal and *H. illucens* larvae meal diets. This suggests that the use of 3% *H. illucens* larvae meal as a replacement for fishmeal in the basal diet did not have the same effect on the colonic immunomodulation in piglets as the addition of 1445 mg zinc/kg zinc oxide to the basal diet. We previously reported that dietary *H. illucens* larvae meal reduced the diarrhea incidence of weaned piglets, but, similarly, without the effect of zinc-oxide-supplemented diets [3]. Overall, dietary *H. illucens* larvae meal can improve the immune homeostasis in piglets and enhance the resistance to ETEC K88 infection when used as a protein feedstuff.

To explore how the possible mechanism of *H. illucens* larvae meal improves the intestinal immune homeostasis, the effect of *H. illucens* larvae meal on histone acetylation modification and the toll-like receptors (TLRs)-NF-κB/MAPK signaling pathway was investigated. Pattern-recognition receptors include TLRs and NOD-like receptors expressed by intestinal epithelial cells and have microbial and pathogen recognition specificity. NOD1 and NOD2 are dependent on a finely orchestrated downstream signaling network, including the NF-κB and MAPK pathways, to regulate the host immune homeostasis [14,15]. TLRs induce the activation of the NF-κB by recruiting the signaling adapter myeloid differentiation factor 88 (MYD88) and proteins containing the TLR structural domain (NF-κB) and mitogen-activated protease (MAPK) pathways linked to signaling molecules to drive the transcriptional expression of pro-inflammatory cytokines [35,36]. The study reports that the early-weaning-induced gut microbial disruption in piglets inhibits autophagy and activates the TLR4/p38MAPK/IL-1β apoptotic signaling pathway, ultimately enhancing intestinal inflammation [37]. In addition, the Shiga toxin and lipopolysaccharide produced by ETEC bind to cell surface receptors and stimulate intestinal epithelial cells and macrophages to activate NF-κB signaling pathways, exacerbating intestinal inflammation [11]. In the present study, dietary *H. illucens* larvae meal increased the relative transcript abundance of NOD2 in the colon, and reduced the activation of the NFκB signaling pathway, similar to the zinc-oxide-supplemented diet. Our previous report showed that dietary *H. illucens* larvae meal reduced the activation of NF-κB and MAPK in the small intestine [3]. Taken together, *H. illucens* larval meal promoted hte colonic immune homeostasis in weaned piglets inhibited the activation of the NF-κB signaling pathway and increased the expression of NOD2.

Elevated levels of the acetylated histones acH3K27, acH3K56, and pH3S10 in the promoter region are typical signs of the transcriptional activation of AMPs. Studies have shown that the inhibition of HDACs increases the pH3S10 expression levels, which, in turn, increases the transcriptional expression of the intestinal β defensin 2 in human colonic epithelial cells and enhances the resistance to ETEC infection [16]. The inhibition of HDACs reduced the macrophage activity and specifically increases the expression levels of the pBD2, porcine β defensin 3 (pBD3), PG1–5, PMAP-37, and PR39 by increasing the level of acH3K9 in the promoter region in the intestines of piglets [38]. In addition, SIRT1 interacts with HDACs to specifically regulate the expression of intestinal AMPs [39]. The transcriptional expression of AMPs is regulated by histone acetylation modifications, and the degree of histone acetylation correlates with the expression levels of HDACs and SIRT1. In this study, we found that *H. illucens*-larval-meal-supplemented diets enhanced the level of histone acetylation mainly through the regulation of acH3K9 and pH3S10 and were associated with a reduced HDAC3 and HDAC7 expression and increased SIRT1 expression. This suggests that there may be a large number of HDAC inhibitors and SIRT1 activators in *H. illucens* larvae that, together, regulate HDACs and SIRT1 to increase the level of histone acetylation. Moreover, SIRT1 plays an important role in maintaining the integrity of the intestinal mucosal barrier [40]. SIRT1 inhibits the activation of the NF-κB/MAPK signaling pathway and is involved in immune regulation [41]. In conclusion, this study reveals the molecular mechanism by which *H. illucens* larvae meal regulates histone acetylation modifications to promote the transcriptional expression of AMPs and thereby improve the immune homeostasis in the colon of weaned piglets.

## 5. Conclusions

In conclusion, the replacement of fishmeal (3% total) with *H. illucens* larval meal improves the colonic immune homeostasis of weaned piglets in ETEC-challenged pig housing. Mechanistically, HILM diets promote antimicrobial peptide expression through increased histone acetylation (acH3K9 and pH3S10).

## Figures and Tables

**Table 1:** Effects of dietary HILM on relative weight of organ of weaned piglets in ETEC-challenged pig housing, %.

Items	NC	PC	HILM	SEM	*p*-Value
Heart weight (g)	106.94	129.23	117.86	3.90	0.105
Liver weight (g)	874.22	863.34	835.50	29.96	0.877
Spleen weight (g)	61.91	67.32	53.92	2.78	0.139
Lung weight (g)	222.59	259.41	248.08	6.87	0.072
Kidney weight (g)	122.44	137.57	129.24	4.22	0.362
Heart weight: BW (%)	0.52	0.55	0.51	0.01	0.221
Liver weight: BW (%)	4.24 ^a^	3.6 ^b^	3.59 ^b^	0.10	0.002
Spleen weight: BW (%)	0.30	0.30	0.23	0.02	0.179
Lung weight: BW (%)	1.08	1.11	1.07	0.02	0.792
Kidney weight: BW (%)	0.59	0.59	0.55	0.02	0.571

NC = basal diet; PC = basal diet supplemented with 1445 mg zinc/kg of zinc oxide; HILM = complete replacement of fishmeal in basal diet with *H. illucens* larvae meal. ^a,b^, Values within a row with different superscripts differ significantly (*p* < 0.05). Data are presented as mean ± SEM; *n* = 6 replicates per treatment.

**Table 2:** Effects of dietary HILM on cytokine production and immunoglobulin in serum of weaned piglets in ETEC-challenged pig housing.

Items	NC	PC	HILM	SEM	*p*-Value
IL-1β ng/L	373.05 ^a^	317.57 ^b^	352.71 ^ab^	9.71	0.050
IL-6 ng/L	59.52	78.30	59.78	4.22	0.110
IL-8 ng/L	34.00 ^a^	29.19 ^a^	13.52 ^b^	3.42	0.001
IL-10 ng/L	51.60 ^b^	65.46 ^a^	63.53 ^a^	2.36	0.022
TNF-α ng/L	171.83	172.10	196.08	21.74	0.886
TGF-β ng/L	146.48	146.34	150.12	8.75	0.851
IgA μg/mL	58.54	63.95	64.02	1.87	0.414
IgM μg/mL	60.65 ^b^	85.00 ^a^	65.5 ^ab^	4.45	0.050
IgG μg/mL	283.09 ^b^	325.14 ^ab^	369.10 ^a^	15.27	0.048

NC = basal diet; PC = basal diet supplemented with 1445 mg zinc/kg of zinc oxide; HILM = complete replacement of fishmeal in basal diet with *H. illucens* larvae meal. ^a,b^, Values within a row with different superscripts differ significantly (*p* < 0.05). Data are presented as mean ± SEM; *n* = 6 replicates per treatment.

## Data Availability

Upon reasonable request, the datasets of this study can be made available by the corresponding author.

## Supplementary Materials

The following supporting information can be downloaded at: https://www.mdpi.com/article/10.3390/ani16010118/s1, Table S1: Ingredient composition of experimental diets (as-fed basis); Table S2: Primers for determination of immunity and barrier function in the colon; Table S3. Antibody information.
