# Supplementary material for: Hermetia illucens Larvae Meal Enhances Colonic Antimicrobial Peptide Expression by Promoting Histone Acetylation in Weaned Piglets Challenged with ETEC in Pig Housing
Source: Animals (Basel). 2025 Dec 31;16(1):118. doi: 10.3390/ani16010118 (PMC12784789; doi:10.3390/ani16010118)
Supplement: Supplementary file 1 [file animals-16-00118-s001.zip › animals-4004948-supplementary.pdf]

Table S1. Ingredient composition of experimental diets (as-fed basis)

Items	NC	PC	HILM
Ingredients (%)			
Corn (8% CP)	28.45	28.45	26.55
Extruded corn (8% CP)	10.00	10.00	10.00
Soybean flour processing (37% CP)	14.00	14.00	14.00
Fermented soybean meal (50% CP)	11.50	11.50	11.50
Soybean meal (46% CP)	7.00	7.00	9.00
Fishmeal (63% CP)	3.00	3.00	-
Hermetia illucens larvae meal (38% CP)	-	-	3.00
Low protein whey powder (3% CP)	15.00	15.00	15.00
Whey protein concentrate (76% CP)	1.00	1.00	1.00
Soybean oil	1.00	1.00	1.00
Sucrose	3.00	3.00	3.00
$NaHPO_4$	0.20	0.20	0.20
NaCl	0.25	0.25	0.25
$CaHPO_4$	0.60	0.60	0.85
Calcium citrate (50%)	1.85	1.85	1.50
L-Lysine-HCl	0.55	0.55	0.55
DL-Methionine	0.15	0.15	0.15
L-Threonine	0.20	0.20	0.20
L-Tryptophan	0.05	0.05	0.05
L-Valine	0.10	0.10	0.10
Choline chloride	0.20	0.20	0.20
TiO_2	0.40	0.40	0.40
Premix [a]	1.50	1.50	1.50
Total	100.00	100.00	100.00
Nutrient levels [b]			
Metabolic energy, $MJ \cdot kg^{-1}$	14.34	14.34	14.35
Crude protein, %	21.03	21.07	20.98
SID-Lysine, %	1.44	1.44	1.43
SID-Methionine, %	0.44	0.44	0.42
SID-Threonine, %	0.85	0.85	0.85
SID-Tryptophan, %	0.26	0.26	0.27
SID-Valine, %	0.92	0.92	0.93
SID-Isoleucine, %	0.76	0.76	0.76
Calcium, %	0.83	0.83	0.80
STTD phosphorus, %	0.35	0.35	0.35
Total phosphorus, %	0.60	0.57	0.54

[a] The premix provides following per kilogram of diet: vitamin A 12 400IU, vitamin D_3 2 800 IU, vitamin E 30 IU, vitamin K 5 mg, vitamin B_1 3 mg, vitamin B_2 10 mg, niacin 40 mg, pantothenic acid 15 mg, folic acid 1 mg, vitamin B_6 8 mg, biotin 0.08 mg, vitamin B_{12} 40 μg, Fe($FeSO_4 \bullet H_2O$)120 mg, Cu($CuSO_4 \bullet 5H_2O$) 16 mg, Mn($MnSO_4 \bullet H_2O$) 70 mg, Zn($ZnSO_4 \bullet H_2O$) 100 mg, I(CaI_2O_6) 0.7 mg, Se(Na_2SeO_3) 0.48 mg.

[b] Crude protein, Ca, and total phosphorus were measured values, whereas the others were calculated values. CP: Crude protein; SID: Standardized ileal digestibility; STTD: Standardized total tract digestible.

Table S2. Primers for determination of immunity and barrier function in the colon

Gene	Sequence (5′-3′)	Size (bp)	Accession number
IL-6	F: TGGCTACTGCCTTCCCTACC<br>R: CAGAGATTTTGCCGAGGATG	132	NM_001252429.1
IL-8	F: TTCGATGCCAGTGCATAAATA<br>R: CTGTACAACCTTCTGCACCCA	176	NM_213867.1
IL-10	F: GCTGAAGACCCTCAGGCTGA<br>R: TTGCTCTTGTTTTCACAGGGC	66	HQ026020.1
IL-22	F: GATGAGAGAGCGCTGCTACCTGG<br>R: GAAGGACGCCACCTCCTGCATGT	112	XM_001926156.1
TGF-β	F: ACGTGGAGCTATACCAGAAATACAG<br>R: ACAACTCCGTGACATCAAAGG	111	NM_214015.1
TNF-α	F: CACGCTCTTCTGCCTACTGC<br>R: GTCCCTCGGCTTTGACATT	164	NM_214022.1
ZO-1	F: AGCCCGAGGCGTGTTT<br>R: GGTGGGAGGATGCTGTTG	147	XM_013993251
Occludin	F: GCACCCAGCAACGACAT<br>R: CATAGACAGAATCCGAATCAC	144	XM_005672525
Claudin-1	F: ACGGCCCAGGCCATCTAC<br>R: TGCCGGGTCCGGTAGATG	221	AJ318102.1
mucin-1	F: ACACCCATGGGCGCTATGT<br>R: GCCTGCAGAAACCTGCTCAT	68	XM_021089730.1
mucin-2	F: CTGCTCCGGGTCCTGTGGGA<br>R: CCCGCTGGCTGGTGCGATAC	100	XM_007465997.1
pBD2	F: CCAGAGGTCCGACCACTACA<br>R: GGTCCCTTCAATCCTGTTGAA	88	AY506573.1
PG1-5	F: GTAGGTTCTGCGTCTGTGTCG<br>R: CAAATCCTTCACCGTCTACCA	273	XM_005669497.2
PR-39	F: CAAGGCCACCTCCGTTTT<br>R: CCACTCCATCACCGTTTTCC	103	NM_214450.1
PMAP-37	F: GCTGTGTGACTTCAAGGAGAA<br>R: GAAATCTCCTGACACCCTCATT	113	NM_001129976.1
NOD1	F: ACCGATCCAGTGAGCAGATA<br>R: AAGTCCACCAGCTCCATGAT	140	NM_001114277.1
NOD2	F: CCTTTTGAAGATGCTGCCTG<br>R: GATTCTCTGCCCCATCGTAG	100	NM_001105295.1
β-actin	F: CACGCCATCCTGCGTCTGGA<br>R: AGCACCGTGTTGGCGTAGAG	380	XM_003124280.4

Table S3. Antibody information

Antibody	Identifier	Source
Anti-MUC2 antibody	ab134119	Abcam
Anti-SIRT1 antibody	ab110304	Abcam
Anti-HDAC7 antibody	ab12174	Abcam
Anti-HDAC3 antibody	ab210688	Abcam
Anti-Histone H3 (acetyl K27) antibody	ab4729	Abcam
Anti-Histone H3 (acetyl K9) antibody	ab4441	Abcam
Anti-Histone H3 (phospho S10) antibody	ab14955	Abcam
Anti-Histone H3 antibody	ab1791	Abcam
Anti-NF-kB p65 (phospho S536) antibody	ab86299	Abcam
Anti-NF-kB p65 antibody	ab32536	Abcam
Phospho-p38 MAPK Antibody	#9211	Cell Signaling Technology
p38 MAPK Mouse Antibody	#9228	Cell Signaling Technology
Anti-TLR2 antibody	ab191458	Abcam
Anti-β-Actin Mouse Monoclonal Antibody	ABL1015	Abbkine
HRP-conjugated Affinipure Goat Anti-Mouse IgG(H+L)	SA00001-1	Proteintech
HRP-conjugated Affinipure Goat Anti-Rabbit IgG(H+L)	SA00001-2	Proteintech
